# Type I Interferon Is a Catastrophic Feature of the Diabetic Islet Microenvironment

**DOI:** 10.3389/fendo.2017.00232

**Published:** 2017-09-14

**Authors:** Brittney N. Newby, Clayton E. Mathews

**Affiliations:** ^1^Department of Pathology, Immunology and Laboratory Medicine, University of Florida, Gainesville, FL, United States

**Keywords:** type 1 diabetes, type 1 interferons, humans, CD8^+^ T cell, beta cells

## Abstract

A detailed understanding of the molecular pathways and cellular interactions that result in islet beta cell (β cell) destruction is essential for the development and implementation of effective therapies for prevention or reversal of type 1 diabetes (T1D). However, events that define the pathogenesis of human T1D have remained elusive. This gap in our knowledge results from the complex interaction between genetics, the immune system, and environmental factors that precipitate T1D in humans. A link between genetics, the immune system, and environmental factors are type 1 interferons (T1-IFNs). These cytokines are well known for inducing antiviral factors that limit infection by regulating innate and adaptive immune responses. Further, several T1D genetic risk loci are within genes that link innate and adaptive immune cell responses to T1-IFN. An additional clue that links T1-IFN to T1D is that these cytokines are a known constituent of the autoinflammatory milieu within the pancreas of patients with T1D. The presence of IFNα/β is correlated with characteristic MHC class I (MHC-I) hyperexpression found in the islets of patients with T1D, suggesting that T1-IFNs modulate the cross-talk between autoreactive cytotoxic CD8^+^ T lymphocytes and insulin-producing pancreatic β cells. Here, we review the evidence supporting the diabetogenic potential of T1-IFN in the islet microenvironment.

## Introduction

Type 1 diabetes (T1D) results from an autoimmune-mediated attack that specifically targets insulin (INS)-secreting pancreatic beta (β) cells. Through the interactions of β cell antigen-specific T cell receptors (TCR) with MHC-peptide complexes, β cells are destroyed leading to aberrant glucose homeostasis and persistent hyperglycemia. Critical to T1D pathogenesis is the targeted destruction of pancreatic β cells mass by autoreactive cytotoxic CD8^+^ T lymphocytes (CTLs) (1–6). Although responses in T1D are directed toward autoantigens, the activation of the β cell specific CTLs is expected to be similar to activation of CD8^+^ T cells observed during a typical response to infectious agents. Following activation, autoreactive CTLs clonally expand, home into the pancreatic islets, and survey the surface of β cells for antigen presented in the context of MHC class I (MHC-I). Recognition of the specific cognate peptide- human leukocyte antigen (HLA) class I complex results in the induction of TCR signaling, formation of the immunological synapse, and targeted destruction of β cells. While the immune system plays a significant role in perpetuating disease pathology, a large body of literature supports the notion that development of T1D is dependent upon a complex network of determinants including those of genetic and environmental etiologies (7–15). Tissue microenvironments influence immune responses in models of tumor biology and infectious disease. However, this notion remains largely been unexplored in the target tissues of autoimmune diabetes.

Type 1 interferons (T1-IFNs), classically known for interfering with viral infection, have been implicated in the early stages of T1D autoimmunity (16–21). Transcriptome analysis reveals a T1-IFN signature in the peripheral blood of patients prior to the development of autoantibodies (16, 17). Additionally, these cytokines have been identified as being expressed in the pancreata of deceased tissue/organ donors with T1D versus non-diabetic donors (18, 19, 21). GWAS studies reveal several T1D-associated genes that are involved in the production, signaling, and regulation of the T1-IFN pathway (12, 22). Moreover, induction of T1D has been reported in patients receiving T1-IFN therapy for various conditions including hepatitis C, multiple sclerosis, and hairy cell leukemia (23–30) supporting the idea that these cytokines may actively exacerbate T1D progression. Despite the growing evidence for the role of T1-IFNs in T1D, little is known about how these cytokines contribute to the inflammatory environment of the human autoimmune diabetic islet (16, 17, 31–36). This review will consider the current paradigms in the natural history of T1D as well as T1-IFN action while summarizing the published literature regarding a role for T1-IFNs in T1D pathogenesis. Additionally, we highlight the exciting new avenues of research suggesting that T1-IFNs may be a catastrophic feature within the diabetic microenvironment.

## Setting the Stage for Autoimmunity: Role of Genetic Susceptibility

Genetic predisposition constitutes a primary risk factor for the initiation of β cell autoimmunity and can be attributed to the complex interplay of more than 50 genetic loci that may impact immune function, INS expression, and β cell function (11, 37, 38). Identified as the first genetic locus associated with T1D in the 1970s, the HLA region on chromosome 6p21 confers approximately 50% of the genetic risk for disease development (39). This region, also referred to as (it) IDDM1 (it), is highly polymorphic, containing over 200 identified genes that can be categorized as class I, II, or III genes that play an important role in antigen presentation as well as regulation of this process. Particularly, class I and II genes encode the classical HLA cell surface proteins that are involved in presenting antigen to CD8+ and CD4+ lymphocytes, respectively. In fact, the strongest association is found in patients harboring the specific HLA class II haplotypes, DR3-DQ2 (DRB*301-DQB*201) and DR4-DQ8 (DRB*401-DQA*301-DQB*302) with the highest risk seen in DR3/DR4 compound heterozygotes (40, 41). Conversely, strong protection from T1D is observed in individuals with the DQB*602 allele, which is reported in less than 1% of patients with T1D (42, 43). Comparison of high- and low-risk DQ alleles in humans and mouse models reveal key differences in peptide binding, as predisposing alleles contain a substitution of non-charged amino acids (alanine, valine, or serine) for aspartate at position 57, which destabilizes binding of antigenic epitopes (44–46). While most studies assessing HLA risk haplotypes have been carried out in Caucasian individuals, recent efforts have begun to characterize HLA susceptibility in other ethnic groups. For example, HLA genotyping in African American patients found that the African-specific DR9 (DRB1*09:01-DQA1*03:01-DQB1*02:01g) haplotype in combination with DR4 mimics risk for T1D seen in patients with DR3/DR4 heterozygosity in European populations. Alternatively, the African-specific “DR3” haplotype (DRB1*03:02-DQA1*04:01-DQB1*04:02) confers significant protection (47). Future studies in this area should be geared toward understanding HLA risk haplotypes in individuals of diverse ethnic backgrounds. Although not as widely studied, HLA class I alleles, HLA A*24 and HLA B*39, appear to be associated with increased susceptibility for T1D, decreased age of onset, and fulminant β cell destruction (48–50).

Numerous additional loci outside of the HLA region summate the remaining genetic risk for diabetes development, although the individual odds ratios conferred by these regions are modest (11, 12). Several of these genes are thought to influence tolerance mechanisms facilitating the escape of autoreactive T cells into the periphery. For instance, variants within the INS gene are known to modulate thymic INS expression, which comprises about 10% of the genetic risk for T1D and carry an odds ratio of 2.2 (51–53). Extensive mapping of this region associates variable number of tandem repeats in the 5′ promoter of INS with diabetes risk (53–55). Shorter class I alleles [23–63 repeats] predispose for diabetes, while longer class III alleles [140–210 repeats] are protective (55). The number of tandem repetitions determines INS transcription in the thymus through interactions with the autoimmune regulator, AIRE, which is essential for appropriate T cell education and provides strong evidence that central tolerance to INS, the primary autoantigen in T1D, is impaired in patients who harbor this risk variant (56).

Protein tyrosine phosphatase non-receptor type 22 (PTPN22) is another well-known example, as this locus confers the third highest genetic association for T1D and is also known to be a regulator of signaling in a variety of immune cell types including lymphocytes, monocytes, dendritic cells (DCs), and neutrophils (57). Case-control and association studies show that this coding variant causes a non-synonymous substitution from an arginine to a tryptophan (R620W) located within the protein-binding domain of PTPN22. Biochemical studies in lymphocytes demonstrate the PTPN22*W620 allele behaves as a gain-of-function mutant with dampened TCR signaling (58). In contrast, the same variant in myeloid derived cell types is highly controversial with some models demonstrating hyper-responsive DC phenotypes with increased T cell activation while others exhibit reduced function and selective impairment of T1-IFN responses following TLR stimulation (59, 60). How might seemingly paradoxical functions be contributing to onset of T1D? On one hand, diminished TCR signaling by the risk variant could impair central and peripheral T cell tolerance, while reduced T1-IFN production by TLRs may hinder effective clearance of β-cell tropic viruses triggering self-reactivity (61). Studies remain ongoing to determine the full gamut of functional consequences induced by this variant.

Like PTPN22, many T1D-associated genes play multiple roles in immune sensing and signaling especially in response to environmental ques, which supports the hypothesis that genetic risk coupled to permissive environmental determinants collectively contribute to diabetes progression. Diabetogenic viruses signify a highly postulated candidate for initiation and potentiation of islet autoimmunity. Critical for the innate immune response to viral infection are T1-IFNs. Several identified genetic loci for T1D also have prominent roles in the induction and signaling of this pathway, including IFIH1 (rs1990760), TYK2 (rs2304256), and STAT4 (rs7574865) (62–64). TYK2 is a tyrosine kinase involved in proximal TI-IFN signal transduction as well as regulation of IFNAR1 surface expression (65–67). Similarly, STAT4 is a key mediator of T1-IFN signaling essential for the generation of Th1 responses, which contribute to the T cell-mediated pathology observed in diabetes (68, 69). Also associated with several other autoimmune disorders, protective variants for each of these genes is associated with reduced T1-IFN signaling (67, 70). IFIH1 encodes the protein MDA5, a cytoplasmic sensor of viral double-stranded RNA. The non-synonymous SNP found in IFIH1 results in alanine to threonine amino acid substitution at position 946 (A946T) and may diminish ATPase activity of MDA5 activity leading to deranged constitutive provocation of T1-IFN as well as blunted viral sensing (62, 71, 72). Compelling evidence in primary human islets reveals that presence of the homozygous risk allele decreases the autonomous innate response to Coxsackievirus B3 (73). Collectively, these data suggest that the A946T risk variant in IFIH1 may act as a double-edged sword, predisposing β cells to persistent enteroviral infection while concurrently promoting deleterious T1-IFN production in and around the islet microenvironment.

## Evolution of Islet Destruction in Human Diabetes

Human β cells act as quintessential metabolic sensors working to integrate environmental cues for rapid and efficient glycemic control (74). Reports of decreased C-peptide responses and reduced glucose tolerance in autoantibody positive individuals suggest that ongoing inflammation precipitates the deterioration of β cell function prior to diabetes onset (75–77). Additionally, β cells are also widely believed to be active participants in promoting a diabetogenic islet microenvironment. For example, MHC-I is known to be hyperexpressed within the islets of T1D patients, suggesting that β cells may be more visible to infiltrating CTL (1, 20, 21, 78). Increasing data insinuates that signals arising from the islet microenvironment, such as T1-IFNs, could trigger such disease promoting adaptations. Additionally, active inflammatory signals within the islet microenvironment prompt substantial variation in the β cell transcriptome and proteome as well as augmenting the capacity for cytokine and chemokine production by islet or β cells (79).

**Figure 1:**
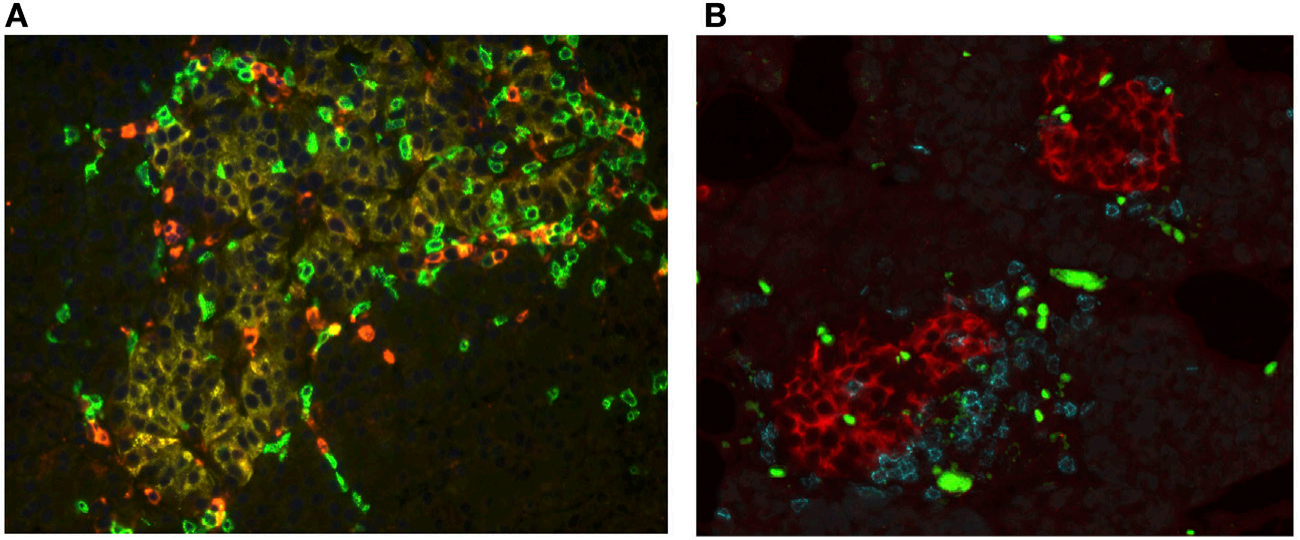
CD8 T cells are the major leukocyte component of the insulitis lesion in type 1 diabetes (T1D) as well as recurrent disease. Pancreatic sections [courtesy of Network for Pancreatic Organ Donors with Diabetes (nPOD)] were examine histologically for the presence of islet invading immune cells. **(A)** Section taken from a pancreas organ donor with T1D (nPOD case 6052). The tissue section was immunofluorescently stained for glucagon (yellow) to identify the islet, CD4 T lymphocytes (red), and CD8 T lymphocytes (green). Within this islet cytotoxic CD8^+^ T lymphocytes (CTLs) are the predominant T cell type observed. Image courtesy of Martha Campbell-Thompson, DVM/PhD (University of Florida). **(B)** Section taken from a pancreas transplant biopsy, from an simultaneous pancreas and kidney (SPK) recipient who had developed recurrence of T1D. Tissues were stained for insulin (red), CD4 T lymphocytes (blue), and CD8 T lymphocytes (teal). Within the insulitic lesions, CTLs represented the dominant T cell found. Figure shows islets with both CD8 than CD4 T cells, but most islets were primarily infiltrated by CD8 T cells. Green/yellow bright stains represent non-specific fluorescence from red blood cells. Image courtesy of Alberto Pugliese, MD, Francesco Vendrame, MD, and George Burke, III, MD, University of Miami.

The conceptual model proposed by George Eisenbarth for the natural history of T1D has shaped theories regarding the evolution of T1D pathogenesis (80, 81). Many facets of this paradigm have been tested and updated over the past 3 decades. The amalgamation of genetic pre-disposition and initiating environmental triggers create the framework for models that describe the insurgence of β cell autoimmunity. Though the nature of the instigating insult is not completely understood, once initiated, active immune-onslaught can be indicated by the presence of autoantibodies and histological detection of the pathognomonic lesion termed insulitis (82). Found in or around the islets, insulitis is a heterogeneous inflammatory infiltrate comprised of T lymphocytes, B lymphocytes, macrophages, and DCs, however CD8^+^ T cells form the primary constituent [(1, 2, 83) and Figure 1A]. First noted by German pathologist Martin Schmidt in the early 1900s, this lesion was not considered a prominent feature of T1D until the landmark paper by Willy Gepts in 1965 where the presence of insulitic lesions were observed in 15/22 recent onset T1D cases (82, 84, 85). Further evaluation of these samples using immunohistochemical techniques and additional data from subsequent studies revealed that inflammation was primarily observed in islets with INS immunoreactivity. Further, in cases with long-standing disease, many islets appear to be devoid of INS containing β cells without active insulitis, alluding to the role of these cells as the inciting antigen in T1D (1, 2, 20, 82, 86–88).

Until recently, efforts aimed at characterizing the nature, composition, and frequency of insulitis have been challenging. This is due to the anatomical inaccessibility of the pancreas for direct study in living subjects as well as a dearth of well-preserved human cadaveric tissues for analysis (84, 89). The inception of the Network for Pancreatic Organ Donors with Diabetes (nPOD) has dramatically advanced our understanding of β cell/Islet autoimmunity (89–92). Moreover, studies of human pancreata have allowed for the emergence of new paradigms in T1D, including the current consensus definition of insulitis, defined as the presence of more than 15 peri- or intrainsulitic CD45^+^ cells within a minimum of three islets (93). The most comprehensive screening and characterization of insulitis to date was recently described using the nPOD collection where a total of 159 pancreata were screened (61 controls, 18 autoantibody positive cases without a diagnosis of T1D, and 80 T1D cases) (88). Investigators presented confirmatory findings that insulitis is present most frequently in recent-onset patients within INS-containing islets and inversely correlates with disease duration. The presence of adaptive-immune infiltration into the islets of individuals with autoantibodies is a rare event, observed only in individuals with multiple antibodies (94). Additionally, patients with T1D display tremendous heterogeneity in terms nature, distribution, and severity of insulitis in addition to the amount of residual β mass present following diagnosis (88, 95).

A critical cell-to-cell interaction during the development of T1D occurs when β cells and islet-antigen specific CTLs come into contact. Strong evidence has supported a crucial role for CD8^+^ T cells in T1D. First reported by Bottazzo in 1985, histological characterization of pancreas sections from T1D cases demonstrated that CTL are the most abundant immune cell type found in human insulitis [(20) and Figure 1A]. Additional studies have confirmed that CD8^+^ T cells have a prominent role in T1D as well as recurrent T1D that occurs after transplantation of islets, pancreas (pancreas alone, or SPK recipients) into patients with T1D. Biopsy and histological examination of the transplanted pancreas demonstrate the accumulation of high numbers of CD8^+^ T cells into INS positive islets in patients who are undergoing active islet autoimmunity (Figure 1B).

Regarded as the final executioner in T1D, CTLs mediate direct β cell destruction through the recognition of epitopes from proteins that are selectively expressed in β cells and are presented by these INS-producing cells the context of MHC-I. Following recognition of cognate antigen, CTLs create a close contact with the target β cell by forming an immunological synapse, where several cytotoxic mechanisms are employed to induce death of β cells. These include the induction of molecules involved in the granule exocytosis pathway such as perforin, granzyme, or granulysin as well as increased surface expression of death ligands such as Fas Ligand and TNF-related apoptosis inducing ligand (96–98). The presence of CTLs specific for well-known autoantigens such as IGRP, preproinsulin, and IA-2, have been documented in islets with augmented MHC-I expression (1, 2, 88). CD8^+^ T cells bearing TCR that are specific for β cell antigens have been detected in circulation of patients. These TCRs imbue CTL with the ability to destroy human β cells *in vitro* (99–102). In patients undergoing recurrent autoimmunity following islet transplantation, autoreactive CD8^+^ T cells are associated with β cell destruction resulting in graft failure (103). This evidence for an essential role of CTL in T1D in humans is further bolstered by studies in mice. Spontaneous diabetes fails to develop in non-obese diabetic (NOD) mice lacking MHC-I or β2 microglobulin (4, 6), while diabetes onset can be accelerated by adoptive transfer of diabetogenic CTL (104, 105).

Mounting evidence suggests that stimuli from the diabetic islet microenvironment likely contribute to autoreactive CTL-mediated β cell cytotoxicity. For example, using NOD adoptive transfer systems with IGRP-specific NY8.3 CD8^+^ T cells, it has been demonstrated that CD8^+^ T cells acquire greater cytolytic capacity and an effector-memory phenotype upon migration into the NOD islet (106–108). As T1-IFNs are linked to increased HLA expression in the pancreatic islets of patients with T1D, suggesting that these cytokines contribute to autoimmune surveillance and promote insulitis. While the effect of T1-IFNs on human islets have only recently begun to emerge, evidence suggests that T1-IFNs are involved in the cross talk between the adaptive immune effectors and the microenvironment of the diabetic islet (16, 17, 31–36, 109, 110).

## Type 1 Interferons

Type 1 interferons belong to a large family of cytokines that were originally described by Alick Issacs and Jean Lindenmann in 1957 as soluble factors responsible for mediating viral interference following a primary virus exposure (111–113). Since then, this large family of cytokines has been further categorized into three distinct classes that play essential roles in cellular-mediated defense against viral and microbial infections as well as in autoimmunity (113–116). Differing in structural homology and signaling receptor complexes, these categories include the T1-IFNs as well as the type 2 interferon [interferon gamma (IFNγ)] and the recently identified type III IFNs including IFNλ1 (IL-29), IFNλ2 (IL-28A), IFNλ3 (IL-28B), and IFNλ4 (114, 117–121). T1-IFNs signal through the heterodimeric IFNAR1-IFNAR2 receptor [IFNAR] and comprises the largest class of IFN including thirteen IFNα subtypes in addition to IFNβ, IFNε, IFNκ, and IFNω. Though multiple T1-IFN subtypes may appear redundant, these distinct entities display unique binding affinities to the IFNAR that result in diverse functional outcomes with respect to antiviral, immunomodulatory, and growth inhibitory activity (122–128). While all T1-IFN subtypes contain several conserved “anchoring” residues that are important for receptor binding, the contribution of residues flanking these anchor points determine the overall binding of these polypeptides to IFNAR1/2 (126–130). As such, IFNβ exhibits the strongest interaction with the receptor out of all T1-IFN subtypes (130).

Type 1 interferons represent an early line of defense against viral infection and can be produced by virtually every cell in the body (131–134). Induction of T1-IFNs are initiated by stimulation of pattern recognition receptors (PRRs) that recognize conserved motifs found on viruses, including toll-like receptors (TLR3, TLR4, TLR7, and TLR9), cytosolic RNA helicases (RIG-I and MDA-5), and cytosolic DNA sensors (131, 133, 134). Following activation of these distinct pathways, the adaptor molecules MAVS (cytosolic RNA sensors), STING (cytosolic DNA sensors), TRIF (TLR3/4), and MyD88 (TLR7/8/9) transduce signals that converge on the activation of TBK-1, which phosphorylates IRF-3 leading to transcription of T1-IFN and IRF-7 that engage in a positive feedback loop for amplification of this response (134–136).

Following production, T1-IFNs signal in an autocrine or paracrine fashion through IFNAR. Engagement of the receptor leads to trans-phosphorylation as well as activation of the tyrosine kinases TYK2 and JAK1 that are constitutively associated with the IFNAR subunits, IFNAR1 and IFNAR2, respectively. Signaling downstream of IFNAR can lead to the activation of several pathways that contribute to the widespread range of effects by T1-IFNs depending upon the cell type and the context in which the TI-IFN signal was received (117, 133, 137, 138). Classically, T1-IFN signaling invokes the activation of STAT1-STAT2 heterodimers that rapidly translocate to the nucleus and complex with IRF9 to form the interferon-stimulated gene factor 3 (ISGF3) complex. Formation of ISGF3 leads to binding of the interferon response element (consensus sequence: TTTCNNTTTC) for the transcription of interferon-stimulated genes (ISGs) that mediate a diverse range of functions (117, 133, 139). Alternatively, T1-IFNs are capable of activating all seven members of the STAT family that can manifest as homodimers or heterodimers to induce downstream signaling and transcription. For instance, T1-IFN induced STAT1 homodimers are known to bind IFNγ activated sequences (GAS; consensus sequence: TTCNNNGAA) to initiate proinflammatory programs similar to IFNγ, whereas T1-IFN induced STAT3 homodimers have been reported to interact with the corepressor complex SIN3A to indirectly counteract inflammatory responses (133, 140–142). Utilization of these alternative T1-IFN signaling pathways is partially determined by the expression of individual STAT family members (143). This concept is clearly evident in lymphocytes. The balance between STAT1 and STAT4 dictates T cell responses following T1-IFN exposure (144). This is highly dependent upon STAT4 expression within the T cell, which is initially induced through activation of TCR signaling. This induces a switch from the “classical” anti-proliferative and proapoptotic actions of STAT1 signaling to STAT4 that promotes T cell proliferation, differentiation, and survival (143–145).

In addition to JAK-STAT signaling, several other non-canonical pathways are known to be induced by T1-IFNs. For example, activation of JAK1 and TYK2 after T1-IFN engagement has been shown to induce the PI3K-AKT pathway that leads to activation of mTOR, which leads to downstream control of protein translation, regulation of cellular division, and proliferation, in addition to activation of IKKβ resulting in NF-κB activity (117, 137). In lymphocytes, the MAPK pathway mediates crosstalk between T1-IFN signaling and the TCR complex resulting in growth inhibition (117). While studies are still ongoing to unmask the complex signaling networks induced by T1-IFNs, the ability of these cytokines to induce a wide array of signaling pathways explains their pleiotropic and sometimes paradoxical biological activities.

Type 1 interferons signaling culminates in the induction of a robust antiviral program. Several key components required for T1-IFN signaling, including STAT1 and IRF9, are also well-known ISGs that act to reinforce and amplify the IFN response. T1-IFNs also act to enhance host defense and pathogen detection by increasing the expression of several PRRs involved in viral sensing, expression of 2,5 oligoadenylate synthetase (OAS) that facilitates eradication viral RNA, as well as upregulation of proteins that interfere at various steps of the viral life cycle, including viral entry, replication, and viral egress from infected cells (146).

Type 1 interferons dynamically regulate the actions of innate and adaptive immune cells, including the ability to enhance NK cell cytotoxicity as well as the production of IL-1β and IL-18 by macrophages (147). These cytokines are also well known for directly and indirectly influencing T cell responses that assist in the eradication of invading pathogens or malignant cells (132, 138, 147, 148). IFNα/β promote the differentiation and maturation of DCs by enhancing the expression of MHC-I and II along with costimulatory molecules (CD40, CD80, CD83, CD86, 4-1BBL) required for efficient CD4^+^ and CD8^+^ T cell priming (138, 149–151). These cytokines promote trafficking of DCs to lymphoid organs, stimulate expression of adhesion molecules, and induce the secretion of chemoattractant molecules that promote communication between DCs and T lymphocytes (138, 152–154). In line with their effects on DCs, T1-IFNs promote the activity of antigen-exposed CD8^+^ T cells, by inciting proliferation, enhancing survival, and increasing effector function. Conversely, in antigen-inexperienced CD8^+^ T cells the T1-IFNs prevent growth and differentiation in an effort to direct a specific T cell response toward the inciting pathogen (138). While T1-IFNs act to implement numerous mechanisms aimed at thwarting the spread of infection, aberrant activation of this pathway, as seen in autoimmunity, can lead to overactivation of immune cells and perpetuation of tissue damage.

## T1-IFNs and Pathogenesis of T1D

### Evidence in Humans

The characterization of insulitis in seminal studies by Gepts and Foulis altered the landscape regarding the pathogenesis of T1D to one of an immune etiology. Soon after, it was reported that there was a striking genetic association between the HLA DR locus and T1D onset (155). These findings, along with the notion that class II antigens could be expressed abnormally in other organ-specific autoimmune diseases prompted investigators to hypothesize that altered antigen presentation by pancreatic β cells in T1D might explain activation and infiltration of autoimmune T cells found within insulitic lesions (156–158). In 1985, Bottazzo et al. reported that residual β cells found in a 12-year-old recent onset donor were selectively positive for HLA-DR. In addition to noting enhanced expression of HLA-DR, this was also the first report to note enhanced HLA class I expression in insulitic islets the same donor (20). Since the aberrant expression of MHC-II molecules could be induced by IFNγ on thyroid follicular cells in autoimmune thyroiditis, it was postulated that IFNγ could be acting in a similar manner to induce this uncharacteristic expression in pancreatic β cells. While subsequent studies showed that interferons were incapable of directly inducing ectopic expression of MHC-II on pancreatic β cells, they were found to be potent inducers of MHC-I expression (21). Subsequent analyses confirmed that MHC-I expression was a prominent phenotype found in patients undergoing islet autoimmunity, especially in normal appearing or inflamed islets containing residual β cells (1, 78, 159–162). Based on the heterogeneity of insulitis in T1D, it was hypothesized that β cells could be actively generating soluble mediators that are capable of acting in a paracrine manner to exert affects within the diabetic microenvironment. IFNα represented a prime candidate, as it was known to induce MHC-I in islet tissue and was known to be produced by a wide range of cells (21). The first report to correlate the presence of IFNα in the islets of patients with recent-onset T1D diabetes was published in 1987. Investigators examined 37 pancreata from cadaveric donors with T1D and found that 34 of 37 samples displayed MHC-I hyperexpression. Further, 97% of patients displaying this feature concurrently exhibited positivity for IFNα by immunocytochemistry (18). Transcript expression of various cytokines, including IFNα, IFNβ, IFNγ, IL-1β, TNFα, were compared in diabetic and control pancreata. Among the panel of cytokines tested, only IFNα displayed a clear and consistent pattern of augmented expression in patients (19).

Additional lines of evidence implicate a pathogenic role for T1-IFNs in human autoimmune diabetes. The presence of β cell-specific autoantibodies signifies the preclinical phase of T1D and serves as an essential biomarker for identifying at-risk individuals (163). Long-term follow up of at risk children enrolled in the BABYDIET and DIPP longitudinal studies reveal T1-IFN inducible signatures in the peripheral blood, which was positively correlated with episodes of upper respiratory infections. The signature was strongest immediately prior to seroconversion and began to decline after the detection of autoantibodies. This time course suggests that activation and production of T1-IFNs may be involved in the early stages of islet autoimmunity (16, 17). In accordance with these findings, IFNα in the plasma of patients with T1D was shown to be elevated when compared to controls (10.1 U/mL; 69.6% positivity vs. 0.4 U/mL, 0% positivity, respectively) and plasmacytoid dendritic cells (pDCs), well known for producing T1-IFNs, were observed in the peripheral blood of new-onset patients during diagnosis (164, 165). Furthermore, enterovirus RNA, particularly Coxsackievirus B, was identified in 50% of patients who displayed positivity for IFNα (165).

The half-life of cytokines within the T1-IFN family is relatively short (IFNα: 4–16 h; IFNβ 1–2 h) and serum levels of IFN begin to decline very rapidly once secreted (166, 167). Due to rapid clearance, detection of IFNs in circulation can prove challenging. To circumvent this, investigators have attempted to use T1-IFN induction pathways, such as Poly(I:C), or the measurement of ISGs in PBMC as surrogate markers for T1-IFN activation when comparing patients and controls (168, 169). For example, patients display a higher basal expression of the ISG OAS, as well as increased sensitivity to IFNα exhibited by maximal induction at lower IFNα concentrations when compared to control subjects (168). T1-IFN production was higher from PBMC isolated from patients with T1D compared to controls, whereas IFNγ production by isolated PBMC in response to concanavalin A was not different between control and T1D patient samples (169). With respect to the IFNα response, there was no correlation to blood glucose levels, HbA1c, age of onset, disease duration, or ICA positivity, which may point to the importance of genes associated with T1D that are involved in signaling of this pathway (169).

Initiation of islet autoimmunity has been noted in individuals following T1-IFN therapy for chronic hepatitis, multiple sclerosis, as well as hematologic malignancies (23, 24). First reported in 1992, T1-IFN-induced autoimmune diabetes was described in a patient with Hepatitis C, who was seropositive before treatment for autoantibodies against both GAD and INS (30). While this complication occurs in a minor subset of patients, half of all cases reporting T1D following IFN therapy were positive for autoantibodies. This suggests that T1-IFNs may precipitate loss of tolerance and self-reactivity in at-risk patients (170). Studies investigating β cell function in these patients suggest that T1-IFNs can reduce INS secretion, impair carbohydrate metabolism during an oral glucose challenge, and induce INS dependency over the course of treatment (171, 172). Patients who incur T1-IFN-induced autoimmune diabetes to not exhibit normoglycemia when T1-IFN therapy is arrested suggesting that in these patients β cell mass is lost to an extent that metabolic control cannot be reestablished.

### Evidence in Animal Models of T1D

Animal models have been indispensable for ascertaining knowledge regarding the cellular and molecular events involved in T1D pathogenesis. Likewise, these models have also been instrumental in elucidating how T1-IFNs contribute to diabetes pathogenesis. One example includes the diabetes prone biobreeding rat (BB-DP rat). These animals emulate some pathologic features observed in human diabetes including polygenic inheritance [including the MHC], peripubescent onset, and β cell destruction characterized by mononuclear infiltration (173, 174). Initial studies conducted in this model demonstrate a dose dependent stimulation of IFNα production by Poly(I:C) that correlates with accelerated diabetes incidence and severity (175, 176). Conversely, elevation of serum IFNα in non-diabetes prone Wistar rats did not instigate diabetes, suggesting that T1-IFNs are not pathogenic without an inherent risk for diabetes (175, 176). Additionally, investigation into the natural history of diabetes in BBDP rats revealed spontaneous expression of IFNα in the islets of Langerhans prior to insulitis proposing that induction of T1-IFNs in the islet microenvironment may disrupt self-tolerance in this preclinical model (177).

The NOD mouse model has served as the principal animal model for the investigation of causative mechanisms leading to autoimmune diabetes (178). Several lines of evidence in the NOD support an association for T1-IFNs in T1D. One of the most striking is the presence of a T1-IFN signature in NOD islets prior to diabetes onset, reminiscent of the signature observed in humans and BBDP rats (31). In 4- to 6-week-old NOD females, T1-IFNs serve as one of the first distinctive signs of pathology in these animals followed by lymphocytic infiltration and synchronized elevation of activation markers in the islet tissue (31). Elevated levels of IFNα and pDC in the pancreatic draining lymph nodes are also reported in 2- to 3-week-old NOD mice (36). This argues that aberrant activation of pDCs, a DC subset that specializes in T1-IFN production, may contribute to the development of this signature, perhaps through ineffective clearance of islet cell debris (36, 179, 180). Moreover, innate sensing by TLRs represents an essential pathway for the stimulation of T1-IFNs. Accordingly, ablation of MDA-5 (encoded by *Ifih1*) in NOD mice results in protection from spontaneous T1D development, while NOD mice carrying a single allele of MDA-5 experience slowed progression and reduced incidence (181). MDA-5^+/−^ animals also displayed protection from Coxsackie B4 virus-induced T1D when compared to MDA-5^+/+^ littermates that developed disease despite being able to efficiently clear the virus (181). Further investigations have revealed that CB4 infection of MDA-5^+/−^ mice resulted in a transient increase in IFNβ that returned to baseline by 7 days postinfection, while IFNβ levels in MDA-5^+/+^ mice remain consistently elevated after infection (181). These data suggest that protective allotypes of MDA-5 may act in a similar manner to tightly regulate IFN production while keeping antiviral defense mechanisms intact (181). Accordingly, stimulation of TLR7, which recognizes ssRNA to promote T1-IFN production, results in accelerated T1D onset in NOD animals, whereas abrogation of TLR9 signaling, important for the response to unmethylated DNA, retards progressive islet destruction (182, 183). Inhibition of T1-IFN signaling through the heterodimeric IFNAR has presented conflicted results. Incidence in NOD and NOD.IFNAR1^−/−^ was indistinguishable, however short-course administration of an IFNAR1 blocking antibody to NOD animals 15–25 postpartum significantly delayed the onset of diabetes (36, 184, 185). Recently, CRISPR-Cas9 deletion of the IFNAR1 subunit in LEW.1WR1 rats, a newly described animal model for T1D, caused delayed onset and frequency of Poly(I:C) induced diabetes (186, 187). Taken together, these data support the idea that coordinated activation of T1-IFN is an early event in autoimmune diabetes but its role in disease progression is likely heavily influenced by the immune response to environmental cues and inheritance of risk/resistance alleles in genes that impact T1-IFN production or signaling.

Utilization of transgenic model systems during the late 20th century provided strong evidence that T1-IFNs may act to accelerate diabetes pathogenesis. Overexpression of IFNα or IFNκ in pancreatic β cells of mice not normally prone to T1D leads to onset of diabetes with severe insulitis, hypoinsulinemia, and diabetes (35, 188). Transgenic mice expressing of IFNβ under the control of the rat INS promoter display various phenotypes depending on genetic background. For example, C57BL6/SJL mice with the RIP-IFNβ transgene do not develop overt diabetes, but display mild hyperglycemia with decreased glucose-stimulated INS secretion and impaired glucose tolerance characteristic of a pre-diabetic state (34). However, overexpression of IFNβ in the islets of other mouse strains that are not prone to developing T1D, including the non-obese diabetes resistant, induced spontaneous diabetes development (34, 189). Moreover, NOD RIP-IFNβ mice had accelerated and fulminant onset of T1D (189). Taken together, these data demonstrate that T1-IFNs can act as a spark leading to autoimmunity but only in individuals that possess an inherent risk for development of T1D. Further, these data demonstrate that T1-IFNs in the islet microenvironment result in deleterious effects on β cell function and viability by promoting islet inflammation.

## T1-IFNs are Major Players in T1D

**Figure 2:**
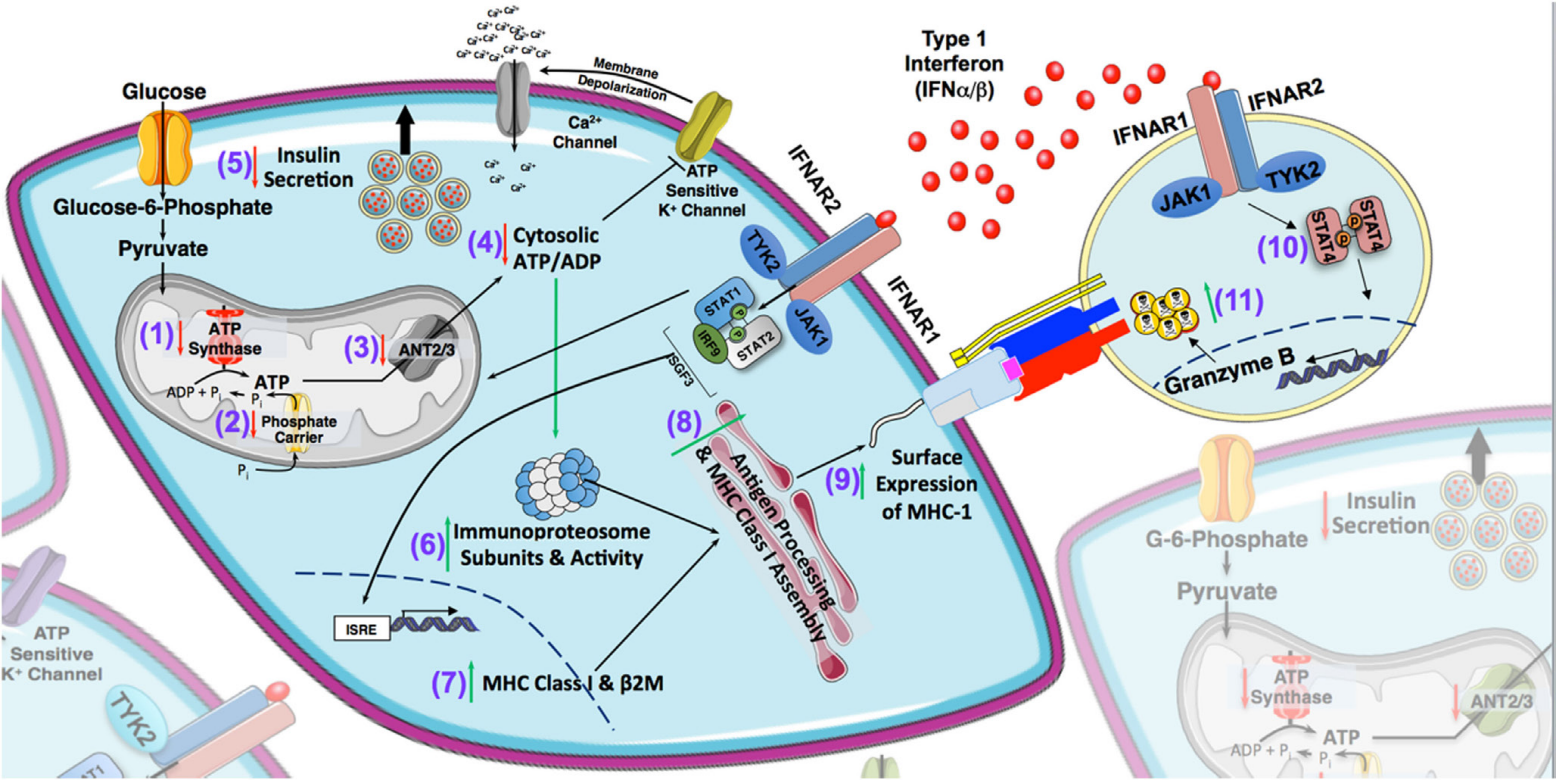
Type 1 interferons (T1-IFNs) are a catastrophic feature of the islet microenvironment in type 1 diabetes (T1D). Based on previous literature and current findings, T1-IFNs are consistently found in the islet autoinflammatory milieu and represent a viable signal that may precipitate diabetogenicity in T1D. With respect to β cells, these cytokines can impair insulin secretory function, possibly through the induction of endoplasmic reticulum (ER) stress as well as by impairing mitochondrial bioenergetics. Whole transcriptome analysis reveals decreased expression of genes involved in the regulation of ATP production and transport, including ATP5A (1), SLC25A3 (2), and SLC25A5/6 (3). Reduction in these transcripts likely lead to decreases in the cytosolic ATP/ADP ratio (4) that is required for glucose-stimulated insulin secretion by β cells (5). T1-IFNs also enhance the autoimmune surveillance of pancreatic β cells through induction of the immunoproteasome (6), *de novo* synthesis of MHC class I and genes responsible for the peptide loading complex (7 and 8), as well as enhanced surface expression of MHC class I (9). This increased capacity for antigen presentation results in a functional ability of cytotoxic CD8^+^ T lymphocyte (CTL)-mediated β cell destruction, which is further augmented by the ability of T1-IFN to amplify infiltrating CTL cytotoxic capacity through STAT4-induced granzyme B production (10 and 11).

Although T1-IFNs have been associated with the induction of T1D and have been identified as a consistent component of the islet autoinflammatory milieu, the direct impact of these cytokines on the pancreatic β cell, cytotoxic T-lymphocytes, and other cellular constituents within the islet that facilitate ongoing islet autoimmunity have only recently been studied using human systems (18, 19, 160, 190). The defining feature observed in T1D is the hyper expression of MHC-I in the islets of patients with T1D, suggesting enhanced β cell immunogenicity and increased susceptibility for targeting by CTLs (1, 2, 18, 19, 190). T1-IFN represent a likely candidate within the local microenvironment capable of mediating this effect, as IFNα/β have been shown to directly induce MHC expression on primary human islet cells [Figure 2 and (21, 109)]. Recent findings by Marroqui et al. demonstrate that IFNα induced HLA is dependent upon canonical T1-IFN signaling, with TYK2, STAT2, and IRF9 being critically required for induction of HLA class I (109). Another notable finding within the islets of new onset T1D patients is elevated levels of the chemoattractant, CXCL10 (191, 192). Touted as a well-known ISG, CXCL10 is induced by IFNα in primary human islets (109). Our laboratory has corroborated these data, showing that exposure of primary islets to T1-IFN results in significant increases in cell surface Class I HLA by flow cytometry as well as increased mRNA expression of MHC-I and CXCL10 by transcriptome analysis (193). Furthermore, we also find upregulation of transcripts critically required for the MHC-I antigen processing and presentation. Enhanced expression of immunoproteasome subunits PSMB8 and PSMB9 (Figure 2) along with proteasome activator subunits PSME1 and PSME2 by T1-IFN suggests an increased efficiency of peptide generation under conditions of inflammatory stress and ATP depletion (194–196). Analysis of constituents of the peptide loading complex following T1-IFN exposure reveal a significant increase in TAP1, TAP2, TAPBP, chaperones, and the editing enzyme ERAP1 suggesting increased transport, stable processing, and loading of peptides onto MHC-I within the endoplasmic reticulum (ER) [(196) and Figure 2]. Additionally, there is a global augmentation of antigen processing and enhanced surface MHC-I with functional reductions in β cell mass, as priming of β cells with T1-IFN results in enhanced CTL-induced lysis by chromium release assay [(193) and Figure 2].

Two recent studies have noted the impact of IFNα on β cells. Using IFNα both groups determined that this cytokine induced the unfolded protein response (UPR) leading to ER stress. However, neither publication reported negative impacts on β cell viability, suggesting that ER stress induced by IFNα did not impact cell death, and there was no reported functional changes (109, 110). While these two studies demonstrate increased expression of markers that signal ER stress, the induction of this response differed in timing and severity, which likely points to differences in experimental design and methodology (109, 110). Indeed, these reports utilized different culture conditions including different media formulations as well as very different time courses of study. For instance, Marroqui et al. revealed an elevated expression of ATF3 and CHOP in primary human islets following 24 h of IFNα (2,000 U/mL) exposure (109). The study conducted by Lombardi and Tomer more widespread induction of the UPR and also assessed INS secretory function in primary human islets and EndoC-βH1 cells after 2 days of exposure to 1,000 U/mL of IFNα. They detected no alterations in glucose stimulated INS secretion, but did correlate the induction of ER stress with reductions in INS content, increased proinsulin to INS ratio, in addition to reduced expression of prohormone convertases, PC1, and PC2 (110). Although ER stress has been a frequently hypothesized explanation for β cell dysfunction in T1D, the idea that IFNα elicits expression of genes involved in the UPR presents a conundrum (197, 198). Previous reports demonstrate that ER stress actually impairs MHC-I expression. These differences in findings of these two recent publications with the discordance of coexisting ER stress and enhanced ER antigen processing highlight the need for a greater understanding of how the numerous signals provoked by T1-IFN alter the β cell in T1D (199–201). Further inspection of metabolic pathways responsible for coordinating INS secretion in β cells by transcriptome analysis revealed a decreased expression ATP5A1, a subunit required for ATP production by ATP synthase; decreased expression of adenine nucleotide translocases 2 and 3 (SLC25A5 and SLC25A6), responsible for regulating mitochondrial ATP export, and decreased expression of the mitochondrial phosphate carrier, SLC25A3 [(193) and Figure 2]. A reduction in these genes will likely have major implications on regulation of glucose-stimulated secretion as they directly alter ATP/ADP ratios that are required to trigger islet cell depolarization that leads to release of INS secretory granules.

Another very important component of the islet microenvironment is vascular endothelial cells that facilitate delivery of oxygen and enable the rapid exchange of nutrients and hormones between the blood and the endocrine pancreas. These cells also act as a barrier to intricately regulate trafficking and extravasation of autoreactive immune cells into the islet microenvironment. Several studies have shown that endothelial cells in and around the islets display an activated phenotype that likely contributes to increased homing and recruitment of autoreactive T cells (202). Immunohistochemical studies examining endothelium within the pancreata of recent onset patients with T1D reveal elevated expression of ICAM-1 as well as hyperexpression of MHC-I and -II (83, 190, 203). Expression of these molecules has also been associated with concomitant expression of IFNα (190). In line with these studies, IFNα is known to directly induce MHC-I and expression of ICAM-1 in endothelial cells, suggesting that these cytokines may increase the capacity for antigen presentation required for autoreactive CTLs to gain entry into the islet (204, 205). Additionally, human pancreatic islet endothelial cells are able to be infected by coxsackievirus B resulting in the production of IFNα, induction of adhesion molecules, and increased interaction with immune cells (206). Mounting evidence suggest that the crosstalk between β cells and the endothelium is important for INS secretory function (207). However, more investigation into the role of T1-IFNs in modulating this interaction is warranted.

It is well known that tissue microenvironments are key determinants in driving local immune responses models of cancer and infectious disease. While armed with the ability to modulate the innate and adaptive arms of the immune system, the impact of T1-IFN within the islet microenvironment has not been fully elucidated. Known to contribute to T cell priming and activation through their effects on DCs, T1-IFNs have been directly shown to mediate DC maturation and migration even in the absence of PPR engagement (208, 209). Specifically, they facilitate the metabolic switch from oxidative phosphorylation to glycolysis through regulation of the transcription factor HIF-1α, inducing the upregulation of MHC-I in these cells as well as costimulatory molecules (208, 209). In the case of the autoimmune diabetogenic microenvironment, the presence of T1-IFNs may act to promote DC immunogenicity skewing toward proinflammatory immune activation in addition to augmenting the function of islet infiltrating immune cells, such as CD8^+^ T cells. Studies completed in our laboratory suggest that T1-IFN drastically augment cytotoxicity elicited by human islet-reactive CTLs. Extensive characterization of T1-IFN signaling mechanisms within these cells show that these cytokines can induce a remarkably rapid acquisition of effector function through induction and direct binding of pSTAT4 to the promoter of Granzyme B (Figure 2). In accordance with studies exhibiting full acquisition of autoreactive CTL effector function within the pancreatic microenvironment, these novel studies implicate T1-IFN as a putative innate signal capable of driving CTL differentiation in the islet (106).

## Conclusion and Model Detailing How IFNα Can Wreak Havoc in the Diabetic Microenvironment

Several determinants predict an individual’s susceptibility to T1D. It is well appreciated that the immune system plays a critical role in diminishing β cell mass, precipitating the onset of persistent hyperglycemia. Critical to this destruction is the presence of CD8^+^ T cells within the diabetic microenvironment. These cells enter the pancreas where they directly target and kill β cells through interactions of the TCR with elevated MHC-I expression on β cells. Soluble factors, such as T1-IFNs, act to promote islet autoimmunity. In addition to being linked to the hallmark HLA class I hyper-expression observed in islets of patients with T1D, T1-IFNs are also well known for their wide-ranging effects including modulation of innate and adaptive immune responses, especially in T lymphocytes. However, until now, few studies to date have focused on elucidating how T1-IFN signaling transforms the islet to an environment that promotes diabetogenicity. The work reviewed here demonstrates that T1-IFNs are stimuli that promote dysfunction and increased visibility of target β cells alongside enhanced CTL effector function leading to β cell destruction.

Association of T1-IFN with T1D reported in previous studies together with our current findings makes a strong case that these cytokines play some role in the complexity of the diabetes puzzle (summarized in Figure 2). It is likely that a genetic pre-disposition skewed toward dysfunctional T1-IFN responses create an islet environment permissive to enhanced autoantigen presentation, augmented human β cell-specific cytotoxicity by autoreactive CTLs and resulting β cell dysfunction. While the pleiotropic actions of T1-IFNs are designed to strengthen the immune response to viral pathogens, this response proves detrimental in the case of autoimmunity where the immune response is misdirected toward self and in this way can promote β cell death in T1D.

## Author Contributions

This manuscript was conceived by BN and CM. Interpretation of data/results and discussion were completed by BN and CM. Manuscript was written and revised by BN and CM.

## Conflict of Interest Statement

The authors declare that the research was conducted in the absence of any commercial or financial relationships that could be construed as a potential conflict of interest.
